# Dimethyl­ammonium 3-carb­oxy­benzoate

**DOI:** 10.1107/S160053681202096X

**Published:** 2012-05-19

**Authors:** Tausif Siddiqui, Vandavasi Koteswara Rao, Matthias Zeller, Sherri R. Lovelace-Cameron

**Affiliations:** a Department of Chemistry, Youngstown State University, One University Plaza, Youngstown, OH 44555, USA

## Abstract

The asymmetric unit of the title organic salt, C_2_H_8_N^+^·C_8_H_5_O_4_^−^, consists of two dimethyl­ammonium cations and two 3-carb­oxy­benzoate anions. The 3-carb­oxy­benzoate anions are linked *via* strong inter­molecular and nearly symmetrical O—H⋯O hydrogen bonds forming infinite chains parallel to [111]. Neighbouring chains are further connected by the dimethyl­ammonium cations *via* N—H⋯O bonds, resulting in a double-chain-like structure. The dihedral angles of all carboxylate groups with respect to the phenylene rings are in the range 7.9 (1)–20.48 (9)°.

## Related literature

For supra­molecular structures comprising 3-carb­oxy­benzo­ates, see: Guo *et al.* (2010 ▶); Liu *et al.* (2007 ▶); Weyna *et al.* (2009 ▶). For similar chain-like structures, see: Ballabh *et al.* (2005 ▶). For hydrolysis of formamides, see: Cottineau *et al.* (2011 ▶); Hine *et al.* (1981 ▶). For a description of the Cambridge Structural Database, see: Allen (2002 ▶). For hydrogen bonding, see: Gilli & Gilli (2009 ▶).
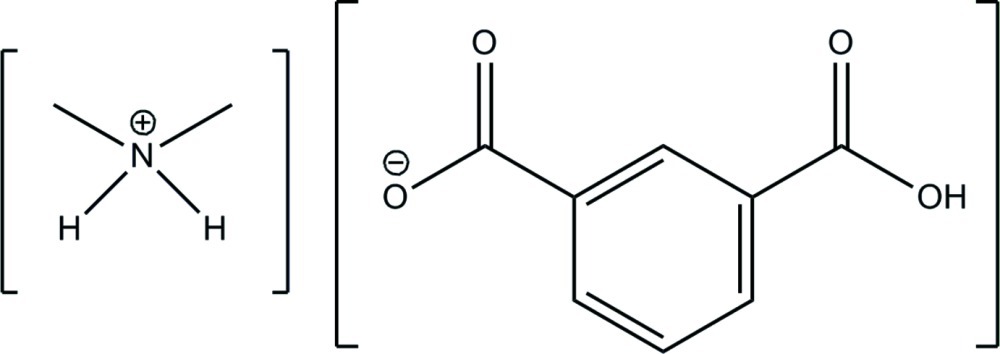


## Experimental

### 

#### Crystal data

C_2_H_8_N^+^·C_8_H_5_O_4_^−^
*M* *_r_* = 211.21
Triclinic, 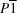

*a* = 8.439 (5) Å
*b* = 10.133 (8) Å
*c* = 12.304 (9) Å
α = 91.858 (14)°
β = 94.599 (17)°
γ = 90.009 (14)°
*V* = 1048.2 (13) Å^3^
*Z* = 4
Mo *K*α radiation
μ = 0.10 mm^−1^
*T* = 100 K
0.32 × 0.21 × 0.09 mm

#### Data collection

Bruker SMART APEX CCD diffractometer
Absorption correction: multi-scan (*SADABS*; Bruker, 2011 ▶) *T*_min_ = 0.684, *T*_max_ = 0.746
13304 measured reflections
6417 independent reflections
4906 reflections with *I* > 2σ(*I*)
*R*_int_ = 0.029

#### Refinement

*R*[*F* ^2^ > 2σ(*F* ^2^)] = 0.049
*wR*(*F* ^2^) = 0.125
*S* = 1.06
6417 reflections
283 parameters
H atoms treated by a mixture of independent and constrained refinement
Δρ_max_ = 0.39 e Å^−3^
Δρ_min_ = −0.29 e Å^−3^

### 

Data collection: *APEX2* (Bruker, 2011 ▶); cell refinement: *SAINT* (Bruker, 2011 ▶); data reduction: *SAINT*; program(s) used to solve structure: *SHELXS97* (Sheldrick, 2008 ▶); program(s) used to refine structure: *SHELXLE* (Hübschle *et al.*, 2011 ▶) and *SHELXL97* (Sheldrick, 2008 ▶); molecular graphics: *DIAMOND* (Brandenburg, 2001 ▶); software used to prepare material for publication: *publCIF* (Westrip, 2010 ▶).

## Supplementary Material

Crystal structure: contains datablock(s) I, global. DOI: 10.1107/S160053681202096X/su2413sup1.cif

Structure factors: contains datablock(s) I. DOI: 10.1107/S160053681202096X/su2413Isup2.hkl

Supplementary material file. DOI: 10.1107/S160053681202096X/su2413Isup3.cml

Additional supplementary materials:  crystallographic information; 3D view; checkCIF report

## Figures and Tables

**Table 1:** Hydrogen-bond geometry (Å, °)

*D*—H⋯*A*	*D*—H	H⋯*A*	*D*⋯*A*	*D*—H⋯*A*
N1—H1*A*⋯O8	0.92	2.00	2.869 (2)	157
N1—H1*B*⋯O2^i^	0.92	1.84	2.744 (2)	166
N2—H2*A*⋯O4^ii^	0.92	1.93	2.822 (2)	164
N2—H2*B*⋯O6	0.92	1.88	2.784 (2)	166
O7—H3*A*⋯O3	1.18 (3)	1.26 (3)	2.4177 (19)	166 (3)
O7—H3*A*⋯O4	1.18 (3)	2.56 (3)	3.329 (2)	121.1 (18)
O5—H5*A*⋯O1^iii^	1.18 (2)	1.27 (2)	2.4483 (19)	171 (2)
O5—H5*A*⋯O2^iii^	1.18 (2)	2.66 (2)	3.462 (2)	124.2 (15)

Symmetry codes: (i) 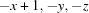; (ii) 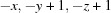; (iii) 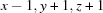.

**Fig. 1.**
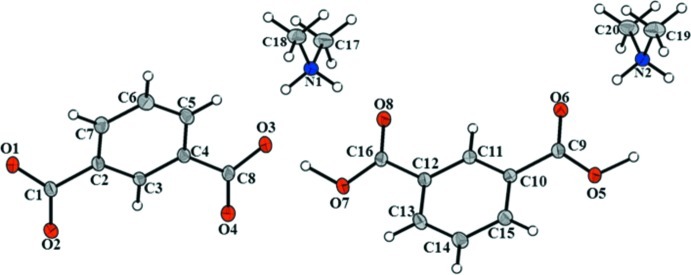
View of the asymmetric unit with the atom-numbering scheme and 50% probability displacement ellipsoids.

**Fig. 2.**
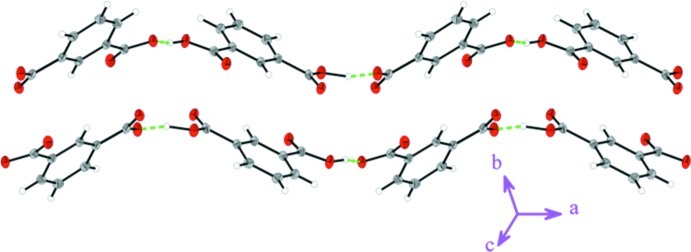
View of the one-dimensional 3-carboxybenzoate chains formed through intermolecular nearly symmetrical O—H···O bonds parallel to the (111) direction.

**Fig. 3.**
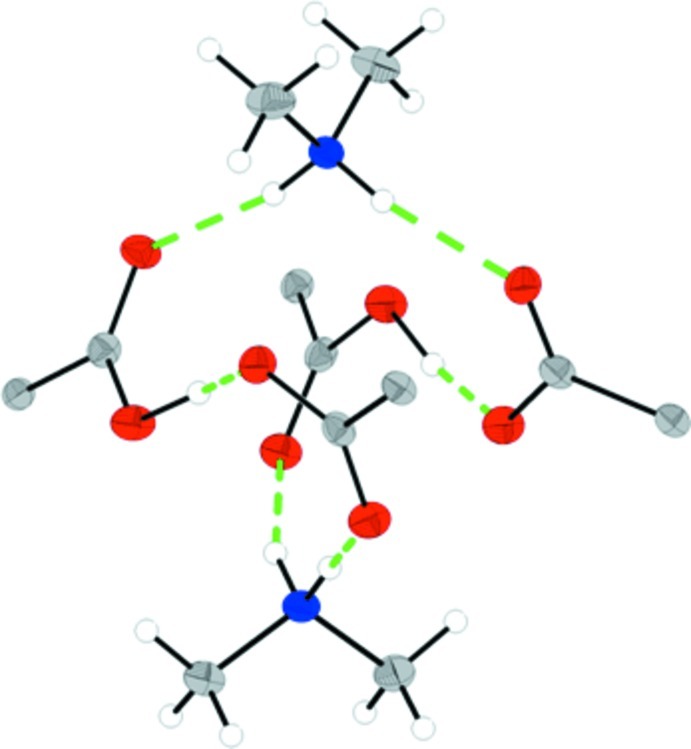
View of one bis(dimethylammonium)—bis(COO-···H+···-OOC) cluster with the dimethylammonium cations bridging between neighboring 3-carboxybenzoate chains through inter-molecular N—H···O bonds. For clarity, only the ipso carbon atoms of the phenylene rings are shown.

**Fig. 4.**
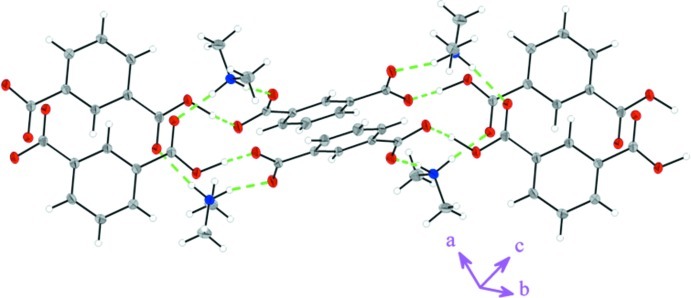
View of the double chain-like structure. A crystallographic inversion center between the two central phenylene rings relates the parallel chains with each other.

## supplementary crystallographic information

### Comment

The title compound, dimethylammonium 3-carboxybenzoate, was obtained as part of our investigations into the solvothermal synthesis of metal organic frameworks of magnesium with aromatic dicarboxylates. Reaction of magnesium nitrate with isophthalic acid and piperazine at 373 K did not yield an extended metal organic framework, but partial decomposition of the DMF solvent let to formation of the title dimethylammonium organic salt, which was isolated as a minor side product in the form of colourless plate-like crystals. Under the rather harsh solvothermal conditions used for the synthesis of many coordination compounds and metal organic frameworks formamides become unstable towards hydrolysis or Lewis acid catalyzed decarbonylation (Hine *et al.*, 1981; Cottineau *et al.*, 2011). This is also evidenced by the high number of dimethyl ammonium salts reported in the Cambridge Structural Database (CSD; Allen *et al.*, 2002; 597 entries up to Feb. 2012), counting only structures that also contain at least one metal ion. With dimethyl amine itself being a gas and being used not extensively as a reagent, it can be safely assumed that most of these structures originated from *in situ* hydrolysis of a dimethyl amide such as DMF. Twenty eight of these entries in the CSD with dimethyl ammonium ions also contain formate ions, the other product of DMF hydrolysis. The title compound, the dimethylammonium salt of isophtalic acid, is one such example that incorporates ammonium cations formed *in situ* through decomposition of a formamide.

The asymmetric unit of the title compound is composed of two hydrogen-3-carboxybenzoate anions and two dimethyl ammonium cations (Fig. 1), which are connected with each other through an intricate network of N—H···O and O—H···O hydrogen bonds (Figs. 2–4). Details of the hydrogen bonding are given in Table 1. Individual 3-carboxybenzoate anions are monoprotonated, and are connected with each other through very strong and nearly symmetric O—H···O hydrogen bonds to form infinite chains parallel to the space diagonal of the unit cell (Fig. 2). Molecules in the chains are arranged in an *ABAB*···fashion, with crystallographically different monoanions alternating with each other. The O—H···O hydrogen bonds are characterized by nearly equidistant D—H and A—H distances (Table 1), as is typical for very strong hydrogen bonds with very electronegative donor and acceptor atoms (Gilli & Gilli, 2009). The keto oxygen atoms of the carboxylate units, which are not involved in the O—H···O hydrogen bonds, act as acceptors for N—H···O hydrogen bonds that originate from both of the dimethylammonium cations, which double bridge the carboxylic acid and carboxylate groups of the anions into a bis(dimethylammonium)—bis(COO^-^···H^+^···^-^OOC) cluster (Fig. 3). In such a manner parallel infinite 3-carboxybenzoate chains are connected into an inversion symmetric double chain like structure (Fig. 4).

Supramolecular structures comprising 3-carboxybenzoates have been reported previously (Guo *et al.*, 2010; Liu *et al.*, 2007; Weyna *et al.*, 2009). Similar one-dimensional chain-like structures have been reported by (Ballabh *et al.*, 2005).

### Experimental

The compound was synthesized under solvothermal conditions. In a typical synthesis, Mg(NO_3_)_2_.6H_2_O (0.064 g, 0.25 mmol) was dissolved in a 1:1 mixture of DMF (5.0 ml) and EtOH (5.0 ml). Then, alumina (Sorbent Technologies, Atlanta, GA) (0.051 g, 0.5 mmol), isophthalic acid (0.166 g, 1.0 mmol) and piperazine (0.043 g, 0.5 mmol) were added to the reaction mixture which was stirred for one hour before transferring the mixture into a glass vial. The final mixture was heated to 373 K for 48 h. The vial was then slowly cooled to room temperature. Slow cooling of the reaction mixture yielded colourless plate-like crystals of the title compound as a minor product.

### Refinement

Hydrogen atoms were placed in calculated positions with C—H bond distances of 0.95 Å (aromatic H), 0.99 Å (methyl H) or 0.88 Å (N—H). Methyl group H atoms were allowed to rotate around the C—C bond to best fit the experimental electron density. Carboxylic acid hydrogen atoms were located in difference electron density maps, but were placed in calculated positions with fixed C—O—H angles, but with the C—C—O—H dihedral angles and the O—H distances freely refined (AFIX 148 command in *SHELXTL* (Sheldrick, 2008)). *U*_iso_(H) values for all H atoms were constrained to a multiple of *U*_eq_ of their respective carrier atom (1.2 times for aromatic and ammonium H atoms, 1.5 times for methyl and carboxylic acid H atoms).

### Crystal data

**Table:**

C_2_H_8_N^+^·C_8_H_5_O_4_^−^	*Z* = 4
*M**_r_* = 211.21	*F*(000) = 448
Triclinic, *P*1	*D*_x_ = 1.338 Mg m^−^^3^
*a* = 8.439 (5) Å	Mo *K*α radiation, λ = 0.71073 Å
*b* = 10.133 (8) Å	Cell parameters from 832 reflections
*c* = 12.304 (9) Å	θ = 2.6–31.1°
α = 91.858 (14)°	µ = 0.10 mm^−^^1^
β = 94.599 (17)°	*T* = 100 K
γ = 90.009 (14)°	Plate, colourless
*V* = 1048.2 (13) Å^3^	0.32 × 0.21 × 0.09 mm

### Data collection

**Table:**

Bruker SMART APEX CCD diffractometer	6417 independent reflections
Radiation source: fine-focus sealed tube	4906 reflections with *I* > 2σ(*I*)
Graphite monochromator	*R*_int_ = 0.029
ω scans	θ_max_ = 31.8°, θ_min_ = 1.7°
Absorption correction: multi-scan (*SADABS*; Bruker, 2011)	*h* = −12→12
*T*_min_ = 0.684, *T*_max_ = 0.746	*k* = −14→14
13304 measured reflections	*l* = −17→18

### Refinement

**Table:**

Refinement on *F*^2^	Primary atom site location: structure-invariant direct methods
Least-squares matrix: full	Secondary atom site location: difference Fourier map
*R*[*F*^2^ > 2σ(*F*^2^)] = 0.049	Hydrogen site location: inferred from neighbouring sites
*wR*(*F*^2^) = 0.125	H atoms treated by a mixture of independent and constrained refinement
*S* = 1.06	*w* = 1/[σ^2^(*F*_o_^2^) + (0.0443*P*)^2^ + 0.3839*P*] where *P* = (*F*_o_^2^ + 2*F*_c_^2^)/3
6417 reflections	(Δ/σ)_max_ < 0.001
283 parameters	Δρ_max_ = 0.39 e Å^−^^3^
0 restraints	Δρ_min_ = −0.29 e Å^−^^3^

### Special details

**Table:**

Geometry. All e.s.d.'s (except the e.s.d. in the dihedral angle between two l.s. planes) are estimated using the full covariance matrix. The cell e.s.d.'s are taken into account individually in the estimation of e.s.d.'s in distances, angles and torsion angles; correlations between e.s.d.'s in cell parameters are only used when they are defined by crystal symmetry. An approximate (isotropic) treatment of cell e.s.d.'s is used for estimating e.s.d.'s involving l.s. planes.
Refinement. Refinement of *F*^2^ against ALL reflections. The weighted *R*-factor *wR* and goodness of fit *S* are based on *F*^2^, conventional *R*-factors *R* are based on *F*, with *F* set to zero for negative *F*^2^. The threshold expression of *F*^2^ > σ(*F*^2^) is used only for calculating *R*-factors(gt) *etc*. and is not relevant to the choice of reflections for refinement. *R*-factors based on *F*^2^ are statistically about twice as large as those based on *F*, and *R*- factors based on ALL data will be even larger.

### Fractional atomic coordinates and isotropic or equivalent isotropic displacement parameters (Å^2^)

**Table:**

	*x*	*y*	*z*	*U*_iso_*/*U*_eq_	
C1	0.59587 (16)	0.02460 (14)	−0.25653 (10)	0.0162 (3)	
C2	0.58993 (16)	0.13501 (13)	−0.17228 (10)	0.0156 (2)	
C3	0.48388 (15)	0.12628 (13)	−0.09138 (10)	0.0146 (2)	
H3	0.4153	0.0519	−0.0910	0.018*	
C4	0.47801 (16)	0.22626 (13)	−0.01100 (10)	0.0155 (2)	
C5	0.57738 (17)	0.33635 (14)	−0.01340 (11)	0.0180 (3)	
H5	0.5745	0.4043	0.0414	0.022*	
C6	0.68042 (18)	0.34716 (14)	−0.09543 (12)	0.0207 (3)	
H6	0.7458	0.4232	−0.0976	0.025*	
C7	0.68748 (17)	0.24595 (14)	−0.17441 (11)	0.0186 (3)	
H7	0.7589	0.2525	−0.2299	0.022*	
C8	0.35934 (16)	0.21816 (14)	0.07316 (11)	0.0171 (3)	
C9	−0.11755 (16)	0.80121 (13)	0.53099 (11)	0.0163 (3)	
C10	−0.11194 (15)	0.71996 (13)	0.42712 (10)	0.0153 (2)	
C11	0.01076 (16)	0.62967 (13)	0.41715 (11)	0.0152 (2)	
H11	0.0899	0.6204	0.4758	0.018*	
C12	0.01843 (16)	0.55276 (13)	0.32181 (11)	0.0156 (2)	
C13	−0.09707 (17)	0.56792 (14)	0.23523 (11)	0.0183 (3)	
H13	−0.0916	0.5167	0.1696	0.022*	
C14	−0.21941 (18)	0.65736 (15)	0.24494 (11)	0.0200 (3)	
H14	−0.2979	0.6673	0.1860	0.024*	
C15	−0.22774 (17)	0.73288 (14)	0.34092 (11)	0.0181 (3)	
H15	−0.3127	0.7934	0.3476	0.022*	
C16	0.15174 (16)	0.45605 (14)	0.31194 (11)	0.0166 (3)	
C17	0.60139 (18)	0.23158 (16)	0.50279 (12)	0.0246 (3)	
H17A	0.5285	0.1660	0.5280	0.037*	
H17B	0.6147	0.3049	0.5567	0.037*	
H17C	0.7048	0.1905	0.4936	0.037*	
C18	0.64148 (18)	0.37914 (15)	0.35102 (12)	0.0230 (3)	
H18A	0.7438	0.3373	0.3395	0.035*	
H18B	0.6586	0.4550	0.4021	0.035*	
H18C	0.5924	0.4091	0.2813	0.035*	
C19	0.07030 (19)	1.03645 (15)	0.80345 (14)	0.0268 (3)	
H19A	0.0896	1.0796	0.8757	0.040*	
H19B	−0.0110	1.0855	0.7601	0.040*	
H19C	0.1691	1.0350	0.7667	0.040*	
C20	0.13291 (19)	0.82098 (16)	0.88063 (13)	0.0262 (3)	
H20A	0.0922	0.7313	0.8874	0.039*	
H20B	0.1530	0.8628	0.9533	0.039*	
H20C	0.2322	0.8171	0.8444	0.039*	
N1	0.53500 (14)	0.28220 (12)	0.39679 (9)	0.0178 (2)	
H1A	0.4386	0.3216	0.4061	0.021*	
H1B	0.5174	0.2123	0.3477	0.021*	
N2	0.01442 (14)	0.89893 (12)	0.81537 (9)	0.0178 (2)	
H2A	−0.0795	0.9006	0.8486	0.021*	
H2B	−0.0050	0.8590	0.7474	0.021*	
O1	0.72388 (12)	0.01992 (10)	−0.30648 (8)	0.0205 (2)	
O2	0.48067 (12)	−0.05209 (10)	−0.27252 (8)	0.0221 (2)	
O3	0.38017 (13)	0.30395 (11)	0.15297 (8)	0.0237 (2)	
H3A	0.255 (4)	0.332 (3)	0.198 (2)	0.092 (10)*	
O4	0.24967 (12)	0.13580 (10)	0.06142 (8)	0.0210 (2)	
O5	−0.24912 (12)	0.86489 (11)	0.53935 (9)	0.0223 (2)	
H5A	−0.260 (3)	0.933 (2)	0.618 (2)	0.066 (8)*	
O6	−0.00266 (12)	0.80375 (11)	0.59990 (8)	0.0212 (2)	
O7	0.13688 (12)	0.38054 (11)	0.22481 (8)	0.0220 (2)	
O8	0.26436 (12)	0.45389 (10)	0.38262 (8)	0.0204 (2)	

### Atomic displacement parameters (Å^2^)

**Table:**

	*U*^11^	*U*^22^	*U*^33^	*U*^12^	*U*^13^	*U*^23^
C1	0.0177 (6)	0.0175 (6)	0.0131 (5)	0.0032 (5)	0.0005 (5)	−0.0018 (5)
C2	0.0163 (6)	0.0160 (6)	0.0140 (6)	0.0037 (5)	−0.0003 (5)	−0.0020 (5)
C3	0.0143 (6)	0.0145 (6)	0.0147 (6)	0.0016 (5)	−0.0001 (4)	−0.0020 (5)
C4	0.0159 (6)	0.0156 (6)	0.0146 (6)	0.0046 (5)	−0.0005 (5)	−0.0014 (5)
C5	0.0211 (7)	0.0142 (6)	0.0181 (6)	0.0023 (5)	−0.0009 (5)	−0.0025 (5)
C6	0.0234 (7)	0.0153 (6)	0.0234 (7)	−0.0017 (5)	0.0016 (5)	−0.0001 (5)
C7	0.0203 (6)	0.0180 (6)	0.0178 (6)	0.0001 (5)	0.0039 (5)	0.0005 (5)
C8	0.0156 (6)	0.0198 (6)	0.0155 (6)	0.0058 (5)	0.0000 (5)	−0.0021 (5)
C9	0.0167 (6)	0.0157 (6)	0.0167 (6)	0.0000 (5)	0.0030 (5)	−0.0015 (5)
C10	0.0147 (6)	0.0164 (6)	0.0148 (6)	−0.0012 (5)	0.0019 (5)	−0.0015 (5)
C11	0.0141 (6)	0.0159 (6)	0.0157 (6)	−0.0005 (5)	0.0015 (5)	−0.0012 (5)
C12	0.0157 (6)	0.0156 (6)	0.0155 (6)	−0.0012 (5)	0.0030 (5)	−0.0016 (5)
C13	0.0227 (7)	0.0189 (6)	0.0129 (6)	0.0006 (5)	0.0011 (5)	−0.0025 (5)
C14	0.0221 (7)	0.0221 (7)	0.0152 (6)	0.0026 (5)	−0.0029 (5)	−0.0001 (5)
C15	0.0174 (6)	0.0185 (6)	0.0183 (6)	0.0023 (5)	0.0008 (5)	−0.0003 (5)
C16	0.0157 (6)	0.0181 (6)	0.0164 (6)	−0.0006 (5)	0.0049 (5)	−0.0026 (5)
C17	0.0206 (7)	0.0306 (8)	0.0221 (7)	−0.0038 (6)	−0.0030 (5)	0.0021 (6)
C18	0.0219 (7)	0.0226 (7)	0.0242 (7)	−0.0064 (6)	0.0000 (6)	0.0001 (6)
C19	0.0229 (7)	0.0225 (7)	0.0340 (8)	−0.0042 (6)	−0.0045 (6)	0.0024 (6)
C20	0.0233 (7)	0.0266 (8)	0.0271 (7)	0.0006 (6)	−0.0080 (6)	−0.0003 (6)
N1	0.0151 (5)	0.0197 (6)	0.0182 (5)	−0.0001 (4)	0.0002 (4)	−0.0036 (4)
N2	0.0156 (5)	0.0219 (6)	0.0156 (5)	−0.0007 (4)	0.0006 (4)	−0.0027 (4)
O1	0.0183 (5)	0.0244 (5)	0.0189 (5)	0.0018 (4)	0.0052 (4)	−0.0058 (4)
O2	0.0196 (5)	0.0229 (5)	0.0231 (5)	−0.0016 (4)	0.0027 (4)	−0.0090 (4)
O3	0.0203 (5)	0.0294 (6)	0.0209 (5)	0.0012 (4)	0.0038 (4)	−0.0111 (4)
O4	0.0194 (5)	0.0249 (5)	0.0188 (5)	−0.0002 (4)	0.0042 (4)	−0.0034 (4)
O5	0.0165 (5)	0.0254 (5)	0.0241 (5)	0.0048 (4)	0.0013 (4)	−0.0095 (4)
O6	0.0171 (5)	0.0276 (6)	0.0179 (5)	0.0029 (4)	−0.0006 (4)	−0.0064 (4)
O7	0.0201 (5)	0.0247 (5)	0.0205 (5)	0.0024 (4)	0.0024 (4)	−0.0099 (4)
O8	0.0179 (5)	0.0213 (5)	0.0214 (5)	0.0043 (4)	0.0006 (4)	−0.0051 (4)

### Geometric parameters (Å, º)

**Table:**

C1—O2	1.2438 (18)	C14—H14	0.9500
C1—O1	1.2850 (17)	C15—H15	0.9500
C1—C2	1.504 (2)	C16—O8	1.2364 (17)
C2—C7	1.395 (2)	C16—O7	1.2945 (17)
C2—C3	1.396 (2)	C17—N1	1.485 (2)
C3—C4	1.3964 (19)	C17—H17A	0.9800
C3—H3	0.9500	C17—H17B	0.9800
C4—C5	1.398 (2)	C17—H17C	0.9800
C4—C8	1.501 (2)	C18—N1	1.485 (2)
C5—C6	1.391 (2)	C18—H18A	0.9800
C5—H5	0.9500	C18—H18B	0.9800
C6—C7	1.394 (2)	C18—H18C	0.9800
C6—H6	0.9500	C19—N2	1.487 (2)
C7—H7	0.9500	C19—H19A	0.9800
C8—O4	1.2427 (18)	C19—H19B	0.9800
C8—O3	1.2915 (18)	C19—H19C	0.9800
C9—O6	1.2354 (17)	C20—N2	1.477 (2)
C9—O5	1.2938 (17)	C20—H20A	0.9800
C9—C10	1.502 (2)	C20—H20B	0.9800
C10—C11	1.392 (2)	C20—H20C	0.9800
C10—C15	1.393 (2)	N1—H1A	0.9200
C11—C12	1.3931 (19)	N1—H1B	0.9200
C11—H11	0.9500	N2—H2A	0.9200
C12—C13	1.397 (2)	N2—H2B	0.9200
C12—C16	1.502 (2)	O3—H3A	1.26 (3)
C13—C14	1.384 (2)	O5—H5A	1.18 (2)
C13—H13	0.9500	O7—H3A	1.18 (3)
C14—C15	1.393 (2)		
			
O2—C1—O1	125.98 (13)	C14—C15—H15	119.9
O2—C1—C2	119.15 (12)	O8—C16—O7	125.23 (13)
O1—C1—C2	114.87 (12)	O8—C16—C12	120.54 (12)
C7—C2—C3	119.73 (12)	O7—C16—C12	114.22 (12)
C7—C2—C1	121.09 (13)	N1—C17—H17A	109.5
C3—C2—C1	119.17 (12)	N1—C17—H17B	109.5
C2—C3—C4	120.34 (13)	H17A—C17—H17B	109.5
C2—C3—H3	119.8	N1—C17—H17C	109.5
C4—C3—H3	119.8	H17A—C17—H17C	109.5
C3—C4—C5	119.39 (13)	H17B—C17—H17C	109.5
C3—C4—C8	119.84 (13)	N1—C18—H18A	109.5
C5—C4—C8	120.69 (12)	N1—C18—H18B	109.5
C6—C5—C4	120.52 (13)	H18A—C18—H18B	109.5
C6—C5—H5	119.7	N1—C18—H18C	109.5
C4—C5—H5	119.7	H18A—C18—H18C	109.5
C5—C6—C7	119.77 (13)	H18B—C18—H18C	109.5
C5—C6—H6	120.1	N2—C19—H19A	109.5
C7—C6—H6	120.1	N2—C19—H19B	109.5
C6—C7—C2	120.22 (13)	H19A—C19—H19B	109.5
C6—C7—H7	119.9	N2—C19—H19C	109.5
C2—C7—H7	119.9	H19A—C19—H19C	109.5
O4—C8—O3	125.16 (13)	H19B—C19—H19C	109.5
O4—C8—C4	120.20 (12)	N2—C20—H20A	109.5
O3—C8—C4	114.61 (13)	N2—C20—H20B	109.5
O6—C9—O5	125.27 (13)	H20A—C20—H20B	109.5
O6—C9—C10	120.41 (13)	N2—C20—H20C	109.5
O5—C9—C10	114.32 (12)	H20A—C20—H20C	109.5
C11—C10—C15	119.48 (12)	H20B—C20—H20C	109.5
C11—C10—C9	119.35 (12)	C17—N1—C18	112.80 (12)
C15—C10—C9	121.16 (13)	C17—N1—H1A	109.0
C10—C11—C12	120.50 (12)	C18—N1—H1A	109.0
C10—C11—H11	119.7	C17—N1—H1B	109.0
C12—C11—H11	119.7	C18—N1—H1B	109.0
C11—C12—C13	119.54 (13)	H1A—N1—H1B	107.8
C11—C12—C16	119.98 (12)	C20—N2—C19	111.54 (12)
C13—C12—C16	120.48 (12)	C20—N2—H2A	109.3
C14—C13—C12	120.12 (13)	C19—N2—H2A	109.3
C14—C13—H13	119.9	C20—N2—H2B	109.3
C12—C13—H13	119.9	C19—N2—H2B	109.3
C13—C14—C15	120.16 (13)	H2A—N2—H2B	108.0
C13—C14—H14	119.9	C8—O3—H3A	114.0 (13)
C15—C14—H14	119.9	C9—O5—H5A	117.8 (12)
C10—C15—C14	120.19 (13)	C16—O7—H3A	115.5 (14)
C10—C15—H15	119.9		
			
O2—C1—C2—C7	−159.33 (13)	O6—C9—C10—C11	11.4 (2)
O1—C1—C2—C7	20.32 (18)	O5—C9—C10—C11	−168.79 (12)
O2—C1—C2—C3	20.12 (19)	O6—C9—C10—C15	−169.00 (13)
O1—C1—C2—C3	−160.23 (12)	O5—C9—C10—C15	10.86 (19)
C7—C2—C3—C4	−1.72 (19)	C15—C10—C11—C12	0.1 (2)
C1—C2—C3—C4	178.82 (12)	C9—C10—C11—C12	179.78 (12)
C2—C3—C4—C5	1.13 (19)	C10—C11—C12—C13	0.8 (2)
C2—C3—C4—C8	177.77 (12)	C10—C11—C12—C16	179.79 (12)
C3—C4—C5—C6	0.5 (2)	C11—C12—C13—C14	−1.0 (2)
C8—C4—C5—C6	−176.10 (13)	C16—C12—C13—C14	−179.93 (13)
C4—C5—C6—C7	−1.6 (2)	C12—C13—C14—C15	0.2 (2)
C5—C6—C7—C2	1.0 (2)	C11—C10—C15—C14	−0.9 (2)
C3—C2—C7—C6	0.7 (2)	C9—C10—C15—C14	179.43 (13)
C1—C2—C7—C6	−179.87 (13)	C13—C14—C15—C10	0.8 (2)
C3—C4—C8—O4	−12.05 (19)	C11—C12—C16—O8	−7.9 (2)
C5—C4—C8—O4	164.55 (13)	C13—C12—C16—O8	171.06 (13)
C3—C4—C8—O3	169.72 (12)	C11—C12—C16—O7	172.93 (12)
C5—C4—C8—O3	−13.68 (18)	C13—C12—C16—O7	−8.10 (19)

### Hydrogen-bond geometry (Å, º)

**Table:**

*D*—H···*A*	*D*—H	H···*A*	*D*···*A*	*D*—H···*A*
N1—H1*A*···O8	0.92	2.00	2.869 (2)	157
N1—H1*B*···O2^i^	0.92	1.84	2.744 (2)	166
N2—H2*A*···O4^ii^	0.92	1.93	2.822 (2)	164
N2—H2*B*···O6	0.92	1.88	2.784 (2)	166
O7—H3*A*···O3	1.18 (3)	1.26 (3)	2.4177 (19)	166 (3)
O7—H3*A*···O4	1.18 (3)	2.56 (3)	3.329 (2)	121.1 (18)
O5—H5*A*···O1^iii^	1.18 (2)	1.27 (2)	2.4483 (19)	171 (2)
O5—H5*A*···O2^iii^	1.18 (2)	2.66 (2)	3.462 (2)	124.2 (15)

Symmetry codes: (i) −*x*+1, −*y*, −*z*; (ii) −*x*, −*y*+1, −*z*+1; (iii) *x*−1, *y*+1, *z*+1.
